# Airport Entry and Exit Screening during the Ebola Virus Disease Outbreak in Sierra Leone, 2014 to 2016

**DOI:** 10.1155/2019/3832790

**Published:** 2019-10-20

**Authors:** Kolitha Wickramage

**Affiliations:** Migration Health Division, International Organization for Migration - United Nations Migration Agency, Migration Health Division, Geneva, Switzerland

## Abstract

We present entry and exit screening outcomes on all persons passing through Freetown International Airport (FNA) in Sierra Leone during the period 1^st^ September 2014 to 4^th^ February 2016. A total of 166,242 persons underwent screening for Ebola Virus Disease (EVD) at FNA. Five persons were denied air travel from Sierra Leone after secondary screening. Laboratory testing revealed none were positive for EVD. No cases were identified through entry screening route. The public health value of airport screening for EVD is discussed.

## 1. Background

On March 17, 2016, World Health Organization (WHO) declared Sierra Leone free of Ebola virus transmission after 42 days (two incubation periods) had passed since the last Ebola patient tested negative [1]. A total of 14,124 cases and 3,956 deaths were confirmed since the epidemic in Sierra Leone occurred in 2014.

Entry and Exit screening is a public health intervention to identify persons with possible symptoms of, or risk of exposure to, EVD, and to prevent them from further travel. On August 8, 2014, WHO under the International Health Regulations (2005) declared the Ebola epidemic in West Africa a Public Health Emergency of International Concern [2]. A recommendation of the emergency committee was that countries with active EVD transmission should conduct exit screening at international airports, seaports, and major land crossings, and that other countries should not generally ban travel or trade [2, 3]. Decision to activate airport screening, however, was made by individual governments. A key benefit of an effective and rigorously conduced airport exit screening is protection of the international community by source containment to prevent international spread [3]. However, there are limited data on screening outcomes from countries with active EVD transmission [4].

Government authorities in Sierra Leone with support from agencies such as the United States Centres for Disease Control (CDC), WHO, and the United Nations Migration Agency (IOM) implemented an EVD screening process for all persons entering and exiting the airport terminal at Freetown International Airport (FNA) in Lungi, Sierra Leone from September, 2014.

## 2. Methods

The key objective of this study was to examine the outcomes of airport entry and exit screening of travellers at FNA during the 2014–2016 West African EVD outbreak. The outcomes for screening modalities in relation to entry and exit screening at FNA are examined in terms of the number of suspected EVD cases detected through primary and secondary screening, suspected cases referred for secondary screening and any cases confirmed via laboratory diagnosis following secondary screening. Cases of EVD in travellers from Sierra Leone that were reported by official government notifications and in the peer-reviewed literature that passed through FNA airport were identified and their health screening forms traced.

### 2.1. Screening Protocols

The screening was undertaken by trained airport staff, including project staff recruited specifically to conduct screening program by IOM, the Sierra Leone Airport Authority, Ministry of Health and Sanitation (MOH) and the Civil Aviation Authority. Secondary screening was supervised by registered medical professionals (medical doctors or nurses), whilst the primary screening was conducted by those with fluency in English language and writing, having previous experience in social or client services sector with a minimum of a diploma. All primary and secondary screening staff were trained by a joint residential training program led by the MOH and IOM with support from the U.S. Centers for Disease Control and Prevention (CDC) and the World Health Organization (WHO). IOM also implemented a Health and Humanitarian Border Management (HHBM) project that began operations on 17^th^ November 2014 to monitor the health screening at FNA [5]. The project contributed 25 primary screening staff that worked on a rotating shift basis to match the 24-hour airport operational times. The project also delivered a training curriculum to over 300 immigration, police, and health personnel, pocket guides on operational procedures based on training content for points-of-entry staff (such as airport personnel, law enforcement and port health officers) and a quality assurance and quality control program in partnership with Sierra Leone Airport Authority and MOH that included simulation exercises on airport incidents (involving actors) to test procedures [6].

Screening steps for travellers implemented were [7]:

1. Notification to travellers that MOHS will be conducting entry and exit screening and that any person who meets risk criteria for EVD infection or exposure may be denied entry into, or exit from, Sierra Leone.
2. Distribution of the Health Declaration Form (HDF) to all prospective travellers. The questionnaire is designed to assist the primary screeners determine whether the traveller meets the risk criteria *(see Appendix A in Supplementary Materials [Supplementary-material supplementary-material-1]for HDF used at Primary Screening*).
3. Traveller screening conducted at FNA to determine the risk of EVD infection or exposure [7]. Risk criteria consist of:
  - Elevated temperature as measured with a noncontact thermometer.
  - Potential for exposure (e.g., caring for ill persons, health care or laboratory worker without using proper Personal Protective Equipment (PPE), and attending funeral for a person with possible EVD).
  - Self-reported or observed signs and symptoms (fever, vomiting, diarrhea headache, red eyes, extreme fatigue, muscle/joint pain, abdominal pain, difficulty breathing, and/or unexplained bleeding).

### 2.2. Primary Screening When Exiting FNA Airport (Figures 1 and 2)

Every departing traveller was required to wash hands on entry to the airport and on entry into the terminal. Departing travellers walked past a thermal camera when entering the terminal, were then requested to complete the HDF in the terminal, and then present the form to the primary screening staff before they are allowed to proceed to check-in. Travellers who answer “no” to symptoms and exposures on the HDF and have recorded temperatures below 38.0°C were be allowed to continue travel. The HDFs were signed and stamped by primary screening staff. Cleared travellers then proceed to check-in counter where the HDF is attached to their boarding pass to indicate that the traveller has undergone screening and is cleared to travel. Another temperature check using a noncontact thermometer was done on all travellers at the boarding gate. If recorded temperature is below 38.0°C, travellers were allowed to board. Travellers who answered “yes” to symptoms or exposures of EVD or who have a recorded temperature 38.0°C and above were referred and directed to secondary screening area for further assessment [7]. Primary screening staff used the Primary Screening Log to indicate the number of passengers screened.

### 2.3. Primary Screening When Entering FNA Airport

HDFs were provided to airlines to distribute in-flight or as soon as they arrive at FNA. Every arriving traveller was required to complete the HDF and present the form to the primary screening staff prior to exiting the immigration area into “Baggage Claim” area. At the screening stations, primary screening staff [7]:

1. Reviewed the HDF for risk factors and symptoms of EVD.
2. Took and recorded the temperature of travellers.
3. Observed travellers for signs and symptoms of illness.

Measures undertaken in event of negative responses on HDF, observations or temperature measures are similar to those mentioned above for exit screening. Every arriving worker to FNA was also required to enter through the main gate in front of the airport, wash hands, and have their temperature taken, and follow the process described above.

Secondary Exit and Entry Screening Procedures for Travellers and Airport staff.

Medically trained staff conducted the Secondary Screening activities upon referral from primary screening points at FNA [7]. Secondary screening staff:

1. Confirmed responses on the HDF for travellers.
2. Complete the Medical Evaluation Record for Sick Traveller or Sick Airport Worker *(see Appendix B in Supplementary Materials [Supplementary-material supplementary-material-1]).*
3. Re-took the traveller's or worker's temperature with a noncontact thermometer.
4. Conducted a visual assessment of traveller or worker.
5. Conducted a detailed public health interview to assess symptoms and exposures using secondary screening form *(see Appendix in Supplementary Materials [Supplementary-material supplementary-material-1])*.
6. Used proper PPE and maintain a distance of 1 metre from traveller or worker (appropriate PPE included: gown, face mask, face shield/goggles, gloves and booties).

Travellers or workers who, in the assessment of the secondary screener, do not meet risk criteria for EVD were allowed to continue travel or work. Those traveller's boarding passes were marked indicating that the traveller has undergone screening. Travellers or workers who meet defined criteria for risk of EVD infection were referred to Ebola Treatment Centre (ETC) at Port Loko or another designated ETC, for appropriate public health or medical interventions after consultation and agreement from the MOHS medical officer. The secondary screener or MOHS Medical officer ensures case referral to the ETC with information about the case being referred. The FNA primary screening staff were requested to notify the ambulance driver that there is a patient to transport to the ETC facility in Port Loko or other designated government ETC hospital. At the time of transport, the screening staff were instructed to notify security to clear the area of everyone not involved with moving the patient to the ambulance. MOHS were responsible for making final determination on clearance to travel or return to work for all travellers and workers who were referred for further testing. MOHS was tasked to determine if travel companions of the suspected ill traveller should be allowed to travel, or are close contacts and must await further evaluation of suspected ill traveller. As per the IHR Committee's recommendations, contacts of Ebola cases were also not allowed to travel internationally until 21 days after exposure, even if asymptomatic.

- Should the ill traveller be cleared for travel by the secondary screener, then the travel companions are also cleared.
- Should the ill traveller be sent for further diagnostic testing, then the travel companions who are close contacts may leave the airport after providing information on how they can be contacted.

Protocol for travellers denied boarding are outlined in [Supplementary-material supplementary-material-1] in Supplementary Materials available here.

### 2.4. Management and Analysis of Airport Screening Data

Data Management and Storage were coordinated by the Port Health staff at FNA in close coordination with IOM. Consent for use of data for analysis and public health decision making were embedded within screening protocol. The signed declaration and consent forms for screening were completed by all travellers as per the regulatory requirement of the Government of Sierra Leone.

Port Health and IOM were responsible to review for consistency and input on a daily basis on the number of HDF forms registered at airline partner agencies (SHP) and from primary screening points. The summary reports and weekly statistics shared included the total number of travellers screened, the number of travellers referred for secondary screening, the number denied boarding and traveller's temperature recordings including mean, mode, and lowest and highest recorded temperatures. The Airport authority (SLAA) Safety and Compliance Officer assured the quality and implementation of the data processes. A detailed description on these data collection, protection, and quality assurance measures are referenced [7]. The data for screening outcomes were entered into a database established by Port Health and IOM. Data for this study were obtained from this database.

A total of 20 confirmed EVD cases were medically evacuated from West Africa to the USA and Europe for treatment [8]. Persons who were medically evacuated from the country were excluded from analysis since they were not part of the routine screening protocols at FNA, as procedures allowed such cases to directly access aircraft carrier designated for evacuation.

## 3. Results

A total of 166,242 persons underwent entry (82,162) and exit (84,080) screening at FNA during the period 1^st^ September 2014 to 4^th^ February 2016. Of those screened, ten cases (0.006%) were identified as being symptomatic or febrile during primary screening. All such cases were captured through exit screening protocols. Of these, five were denied travel and referred for further clinical evaluation following secondary screening by a medical officer at airport. All five (3 foreign and 2 Sierra Leone nationals) were confirmed to be positive for Malaria Falciparum via smear microscopy, and two were co-infected with Typhoid. The remaining five were diagnosed as having mild upper respiratory tract disease. None of the ten were health care workers nor had any history of contact with EVD cases, their known contacts or ETCs. Entry screening did not detect any case for secondary screening.

All 10 persons referred for secondary screening were detected by the hand-held NCIT at health screening posts. No referrals were made from the fixed thermal scanners that were set up at entry and exit points of airport.

Two persons (a British and an Italian) that acquired EVD in Sierra Leone and diagnosed with EVD after developing symptoms at destination country passed through exit screening at FNA. The health screening forms for both cases were traced. Both travellers were health workers who had declared their clinical work with EVD patients. Temperature measurement and symptomology at time of screening had not warranted travel restriction.

## 4. Discussion

WHO recommended exit screening by the EVD affected countries to curtail international spread. This study aimed to assess airport entry and exit screening outcomes in a country with active EVD transmission, and found exit screening detected a relatively low-yield of cases screened at airport with symptomology and self-reported transmission risks relevant to EVD. Entry screening at points of entry undertaken as an additional measure prove ineffective as a screening modality.

The low yields for exit screening are consistent with those entry screening outcomes for EVD in airport settings of high-income nations [9–14]. Of 1,993 arrivals to the United States of America from countries with EVD transmission (during October 2014 to November 2014), a total of 86 travellers (4.3%) were referred to public health officers—all of whom were health care workers [11]. None were diagnosed with Ebola. In New South Wales, Australia between 1^st^ October 2014 and 13^th^ April 2015, public health staff assessed a total of 122 travellers arriving from countries with EVD outbreaks [14]. Six people (5%) developed symptoms compatible with EVD and required further assessment, none developed EVD. State health authorities recommended targeted monitoring of at-risk populations such as returning health care workers. In the UK, 3388 passengers were screened at airports between 14 November 2014 and 4 January 2015, and 130 people were referred for follow up with no cases of EVD reported [14]. A European CDC review concluded that entry screening has an exceedingly low yield, contributing to a limited extent to the prevention of importation of EVD disease [10]. Screening of other infectious disease at points of entry have also yielded low detection rates [15, 16].

Airport exit screening has the potential to miss asymptomatic infected persons or those who fail to declare their exposures. Passengers aware of presenting with fever may be tempted to conceal it for fear of being prevented from boarding a flight or entering the country by using antipyretic drugs. Prevalence of other febrile infectious diseases among travellers in West Africa such as malaria and typhoid are also high. The positive predictive value of a positive screening result based on fever is small as shown by research in the context of pandemic influenza screening based on point of entry screening [17, 18].

Temperature monitoring is a key element of the screening protocol, and may account for detection thresholds. Studies have indicated NCITs to have sensitivities of 80–99%—indicating that between 1% and 20% of the febrile passengers will not be detected (false negative) [19]. Specificity of NCITs are reported at 75–99%, where between 1% and 25% of nonfebrile passengers will be false positive [19]. Advanced infection screening systems for airports are available today that utilize neural network-based platforms [20, 21]. These integrative technologies combine heart rate measures (through a reflective photo sensor), noncontact respiration radar and ear temperature (measured by a thermography) to achieve greater sensitivity than facial skin temperature systems. These may, however, be significantly more expensive, require more advanced training and more frequent calibration [21].

## 5. Conclusion

This study assessed entry and exit EVD screening outcomes for travellers in a country undergoing active EVD transmission. The screening measures implemented at FNA required a high degree of coordination by multiple stakeholders at airport setting ranging from health, immigration, border control, retail, and airline staff. Exit screening, as recommended by WHO emergency committee for countries such as Sierra Leone, resulted in a very low detection yield for suspect cases. However, entry screening for countries with active EVD transmission may not be an effective strategy.

A major aim of screening is the opportunity to identify travellers at risk of later developing EVD, providing health education and ultimately guidance on any follow-up measures with public health authorities if symptoms do manifest [11]. Existence of exit screening may also deter some people with high risk contacts or fever from attempting to travel. Screening measures are best coupled with health promotion programs to sensitize public on EVD disease prevention and build capacities in infection prevention and control for airport staff.

Both known cases of travellers who contracted EVD in Sierra Leone, remained asymptomatic during exit screening process were health care workers in direct contact with EVD patients. Encouraging close monitoring of healthcare workers in countries of destination that have been in contact with EVD patients is therefore a critical strategy [10–12, 14].

Finally, studies involving mathematical simulation models support exit screening at international points of departure, rather than entry screening of all flights arriving directly from affected countries [4]. However, the estimated exit screening detection thresholds are exceedingly high in such models. Findings from this paper may better guide future modelling studies.

## Figures and Tables

**Figure 1:**
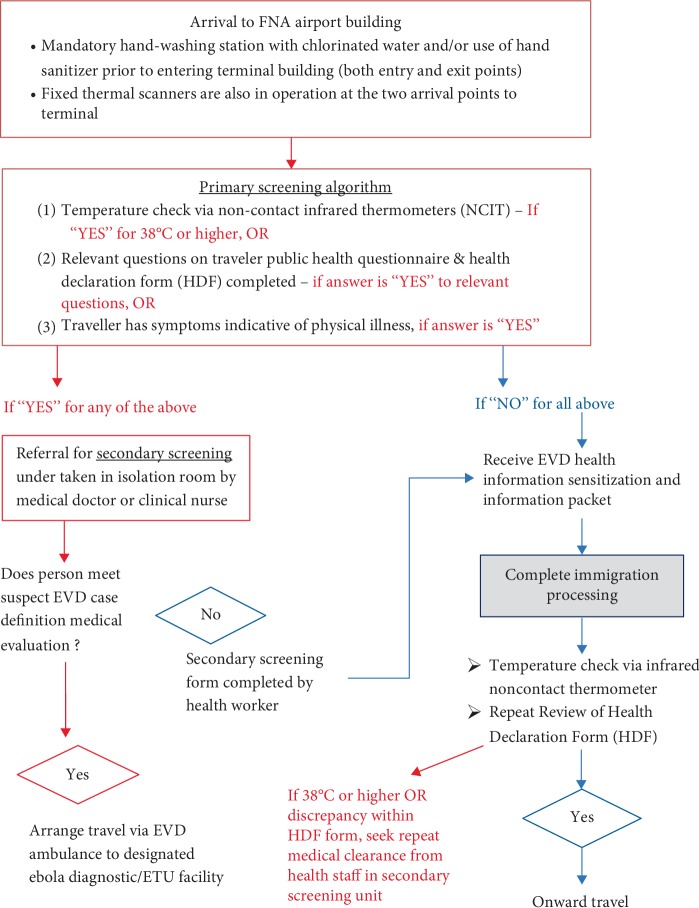
Overview of primary and secondary screening algorithm.

**Figure 2:**
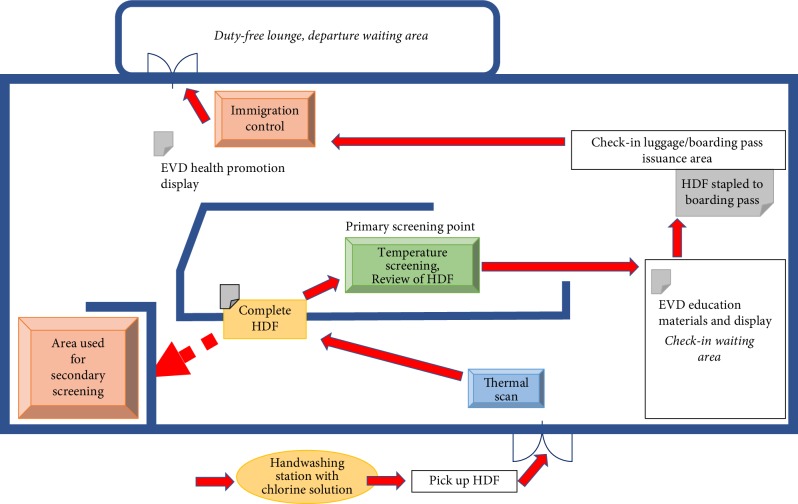
FNA exit screening process.

## Data Availability

The data for screening outcomes were obtained from database maintained by the Port Health Authority of the Ministry of Health and Sanitation (MOHS) which can be requested by the health authority. The resources utilized to construct the screening algorithms described in paper are available from the corresponding author upon request.

## Supplementary Materials

Supplementary Materials Appendix A: health Declaration Form (Primary Screening Form). Appendix B: secondary Screening Form. Appendix C: protocol for travellers denied boarding [7].
