# Supplementary material for: Airport Entry and Exit Screening during the Ebola Virus Disease Outbreak in Sierra Leone, 2014 to 2016
Source: Biomed Res Int. 2019 Oct 20;2019:3832790. doi: 10.1155/2019/3832790 (PMC6930773; doi:10.1155/2019/3832790)
Supplement: Supplementary Materials — Appendix A: health Declaration Form (Primary Screening Form). Appendix B: secondary Screening Form. Appendix C: protocol for travellers denied boarding [7]. [file 3832790.f1.pdf]

Appendix 1: Health Declaration Form (Primary Screening Form)

Republic of Sierra Leone

**Airline:**		**Temperature of Traveler (◦C):**	
**Flight Number:**		**Date of Interview (DD/MM/YYY):**	

HEALTH DECLARATION FORM: FORMULAIRE DE DECLARATION SANTE

This form is to be used to obtain important information from passengers entering or leaving the country, through the Freetown International Airport, on any possible exposure to the Ebola virus. The data received through this form will be treated confidentially in accordance with the International Health Regulations.

**A. Passenger Information**	
1. Name of Passenger:	8. Permanent Address:
2. Gender:<br>3. Age:	9. Address in Sierra Leone:
4. Date of Birth (DD/MM/YYYY):	10. Telephone in Sierra Leone:
5A. Country flight is **going to:**	11. Mobile phone (include country code):
5B. Country flight is **coming from:**	12. E-mail address:
6. Nationality:	13. Countries visited in last 2 months, including Sierra Leone:
7. Passport Number:	14. Areas you visited in that country:
	**Temperature of Traveler at Boarding Gate (◦C):**

**B. Exposure Information – In the past 3 weeks:**	**Yes**	**No**
**Did you have any close contact with a sick person with fever and bleeding?**	◻	◻
If yes, did you wear appropriate personal protective equipment (PPE) during all exposures?	◻	◻
If yes, did you experience any breaches in PPE?	◻	◻
**Did you work in a laboratory processing body fluids of Ebola patients?**	◻	◻
If yes, did you wear appropriate PPE and follow standard lab biosafety precautions at all times?	◻	◻
If yes, did you experience any breaches in PPE or biosafety?	◻	◻
Did you attend a funeral?	◻	◻
Were you hospitalized?	◻	◻
Name of hospital		
Reason for hospitalization		

**C. Health Status- Now or in the last 2 days have you had:**	**Yes**	**No**
Fever	◻	◻
Headache	◻	◻
Vomiting/nausea	◻	◻
Diarrhea	◻	◻
Intense fatigue	◻	◻
Difficulty breathing	◻	◻
Muscular/joint pain	◻	◻
Abdominal pain	◻	◻
Redness of eyes	◻	◻
Unusual bleeding	◻	◻

Passenger's Signature:

FOR OFFICIAL USE ONLY

**D. Screening Outcome (to be completed by screener)**	**Yes**	**No**
**Allowed to board flight**	◻	◻
**Referred to further medical assessment**	◻	◻
Time referred to further medical screening	◻	◻
**Public health follow up in hospital**	◻	◻
**Public health follow up in location other than hospital**	◻	◻
List location:		
**Referred for secondary screening**	◻	◻

Health Officers Comments, Signature, and Stamp

Appendix 2: Secondary Screening Form

Freetown International Airport, Sierra Leone

Date of Interview ______

Time of Interview ______

Republic of Sierra Leone

PUBLIC HEALTH INTERVIEW: SECONDARY SCREENING

Reason Individual Has Been Referred for Secondary Screening -
check all that apply & attach primary screening form (health declaration form)

☐ Symptom(s) ☐ Exposure(s)

SECTION 1: TRAVELER INFORMATION

Individual's Surname: ________________________ Other Name(s): _________________________

Passport #: ____________________________ Passport Country: __________________________

Emergency Contact: _________________________

Location Where Traveler Either Had Exposure or Became Ill:

Village/Town: _______________ District: _______________Province:_____________________

If different from permanent residence, dates residing at this location:
___/___/_____ (DD/MM/YYYY) to ___/___/_____ (DD/MM/YYYY)

SECTION 2: EXPOSURES AND RISK FACTORS – to ask of the traveler

In the last 3 weeks:

**Did you have close contact with a known or suspect case of Ebola, or with any sick person?**	□ Yes	□ No	□ Unk
If yes, did you wear appropriate personal protective equipment (PPE) during all exposures?	□ **Yes**	□ **No**	□ **Unk**
If yes, did you experience any breaches in PPE?	□ **Yes**	□ **No**	□ **Unk**
**Did you work in a laboratory processing body fluids of Ebola patients?**	□ **Yes**	□ **No**	□ **Unk**
If yes, did you wear appropriate PPE and follow standard lab biosafety precautions at all times?	□ **Yes**	□ **No**	□ **Unk**
If yes, did you experience any breaches in PPE or biosafety?	□ **Yes**	□ **No**	□ **Unk**
**Did you attend a funeral?**	□ **Yes**	□ **No**	□ **Unk**
**Did you travel outside your home or village/town?**	□ **Yes**	□ **No**	□ **Unk**
**Were you hospitalized, or did you go to a clinic or visit anyone in the hospital?**	□ **Yes**	□ **No**	□ **Unk**
If yes, name of hospital or clinic:			
If yes, reason for hospitalization or visit:			
**Did you consult a traditional/spiritual healer?**	□ **Yes**	□ **No**	□ **Unk**
**Did you have direct contact (hunt, touch, eat) with animals or uncooked meat?**	□ **Yes**	□ **No**	□ **Unk**

Date of Exposure (If Applicable): ____/____/________ (DD/MM/YYYY)

SECTION 2: CLINICAL SIGNS AND SYMPTOMS – to ask of the traveler

Have you taken fever- or pain-reducing medicine in the last 4 hours? □ Yes □ No

Have you experienced any of the following symptoms today OR within the past 48 hours?

**Fever (≥38.0° C)**	□ Yes	□ No	□ Unk
If yes, Onset date ___/___/____ (DD/MM/YYYY) ***Temp:____° C***			
**Vomiting/nausea**	□ Yes	□ No	□ Unk
**Diarrhea**	□ Yes	□ No	□ Unk
**Intense fatigue/general weakness**	□ Yes	□ No	□ Unk
**Anorexia/loss of appetite**	□ Yes	□ No	□ Unk
**Abdominal pain**	□ Yes	□ No	□ Unk
**Chest pain**	□ Yes	□ No	□ Unk
**Muscle pain**	□ Yes	□ No	□ Unk
**Joint pain**	□ Yes	□ No	□ Unk
**Headache**	□ Yes	□ No	□ Unk
**Cough**	□ Yes	□ No	□ Unk
**Difficulty breathing**	□ Yes	□ No	□ Unk
**Difficulty swallowing**	□ Yes	□ No	□ Unk
**Sore throat**	□ Yes	□ No	□ Unk
**Jaundice (yellow eyes/gums/ skin)**	□ Yes	□ No	□ Unk
**Conjunctivitis (red eyes)**	□ Yes	□ No	□ Unk
**Skin rash**	□ Yes	□ No	□ Unk
**Hiccups**	□ Yes	□ No	□ Unk
**Pain behind eyes/sensitive to light**	□ Yes	□ No	□ Unk
**Coma/unconscious**	□ Yes	□ No	□ Unk
**Confused or disoriented**	□ Yes	□ No	□ Unk

**Unexplained bleeding from any site**	□ Yes	□ No	□ Unk
***If yes*, please specify: __________________________ ____**			
**Bleeding of the gums**	□ Yes	□ No	□ Unk
**Bleeding from injection site**	□ Yes	□ No	□ Unk
**Nose bleed (epistaxis)**	□ Yes	□ No	□ Unk
**Blood or black stools (melena)**	□ Yes	□ No	□ Unk
**Fresh/red blood in vomit (hematemesis)**	□ Yes	□ No	□ Unk
**Coughing up blood (hemoptysis)**	□ Yes	□ No	□ Unk
**Bleeding from vagina, other than menstruation**	□ Yes	□ No	□ Unk
**Bruising of the skin (petechiae/ecchymosis)**	□ Yes	□ No	□ Unk
**Other hemorrhagic symptoms**	□ Yes	□ No	□ Unk
***If yes*, please specify: __________________________ ____**			
**Other non-hemorrhagic clinical symptoms**	□ Yes	□ No	□ Unk
***If yes*, please specify: __________________________ ____**			

SECTION 4: TRIAGE, RESPONSE, CLINICAL SPECIMENS AND LABORATORY TESTING

Please record the outcome of your assessment:

**Travel Intervention**:	**Medical Assessment and Intervention (check all that apply)**:
☐ Allowed to board flight<br><br>☐ Not allowed to board flight<br>Decision made by:<br>☐ Port Health<br>☐ Airline<br>☐ Other, list___________<br>__________________________________<br>__________________________________<br>__________________________________	☐ Further assessment by surveillance officers<br>☐ Held at airport for observation<br>☐ Transported to government hospital/healthcare facility<br>☐ Referred to medical provider of choice for further evaluation to clear for travel<br>☐ Laboratory specimen taken and sent to laboratory for testing for Ebola<br>☐ Suspect Ebola patient (complete travel companion listing form)<br>☐ Illness not related to Ebola<br>☐ None<br>☐ Other, specify<br>____<br>____

Appendix 3: Protocol for travellers denied boarding [7]

Travellers Denied Boarding

If a traveller is denied boarding, FNA were mandated to notify (verbally and in writing) the traveller as well as the airline responsible and SLAA authorities provide safe transportation of the ill traveller to ETC for further public health evaluations. Secondary screening staff retained the HDF and Secondary Screening forms of all travellers who were referred to secondary screening or denied travel. Copies of the HDF and secondary screening form are retained by airport authority, CDC, Port Health authority and original HDF and secondary screening form to IOM.

Clearance to Travel after ETC Evaluation

Individuals denied boarding because they are ill required further assessment and medical clearance to be allowed to travel once the ETC provides diagnostic evidence that laboratory tests are negative for EVD. If a diagnosis of a serious condition other than EVD is made, then a medical professional was required to certify that the ill traveller is fit to fly. Persons with fever only may be allowed to travel after 48 hours provided that the fever resolved and that vomiting, diarrhea or unexplained bleeding did not develop during that time period. Persons who were denied boarding because of vomiting, diarrhea or unexplained bleeding but who did not have a fever may leave after 24 hours provided that they are well do not have a fever, vomiting, diarrhea, or unexplained bleeding. Individuals denied boarding because of high risk exposures 21 days or less before travel were reported to the DSO. These individuals need to be followed for 21 days since the last exposure before being allowed to fly.
